# Fractal-Based Analysis of Bone Microstructure in Crohn’s Disease: A Pilot Study

**DOI:** 10.3390/jcm9124116

**Published:** 2020-12-20

**Authors:** Judith Haschka, Daniel Arian Kraus, Martina Behanova, Stephanie Huber, Johann Bartko, Jakob E. Schanda, Philip Meier, Arian Bahrami, Shahin Zandieh, Jochen Zwerina, Roland Kocijan

**Affiliations:** 1 1st Medical Department, Hanusch Hospital, 1140 Vienna, Austria; judith.haschka@osteologie.lbg.ac.at (J.H.); daniel.arian.kraus@gmail.com (D.A.K.); huberstephy@yahoo.com (S.H.); johann.bartko@oegk.at (J.B.); jochen.zwerina@osteologie.lbg.ac.at (J.Z.); 2 Ludwig Boltzmann Institute of Osteology, Hanusch Hospital of OEGK, AUVA Trauma Center Vienna-Meidling, 1140 Vienna, Austria; martina.behanova@osteologie.lbg.ac.at; 3 Department of Trauma Surgery, AUVA Trauma Center Vienna-Meidling, 1120 Vienna, Austria; jakob.schanda@gmail.com; 4 Ludwig Boltzmann Institute for Experimental and Clinical Traumatology, 1200 Vienna, Austria; 5 ImageBiopsy Lab., 1140 Vienna, Austria; p.meier@imagebiopsy.com; 6 Department of Radiology and Nucelar Medicine, Hanusch Hospital Vienna, 1140 Vienna, Austria; arian.bahrami@oegk.at (A.B.); shahin.zandieh@oegk.at (S.Z.); 7 Medical Faculty of Bone Diseases, Sigmund Freud University, 1020 Vienna, Austria

**Keywords:** Crohn’s disease, bone microstructure, bone loss, fractal-based analysis, glucocorticoid treatment, risk factors, imaging methods

## Abstract

Crohn’s disease (CD) is associated with bone loss and increased fracture risk. TX-Analyzer™ is a new fractal-based technique to evaluate bone microarchitecture based on conventional radiographs. The aim of the present study was to evaluate the TX-Analyzer™ of the thoracic and lumbar spine in CD patients and healthy controls (CO) and to correlate the parameters to standard imaging techniques. 39 CD patients and 39 age- and sex-matched CO were analyzed. Demographic parameters were comparable between CD and CO. Bone structure value (BSV), bone variance value (BVV) and bone entropy value (BEV) were measured at the vertebral bodies of T7 to L4 out of lateral radiographs. Bone mineral density (BMD) and trabecular bone score (TBS) by dual energy X-ray absorptiometry (DXA) were compared to TX parameters. BSV and BVV of the thoracic spine of CD were higher compared to controls, with no difference in BEV. Patients were further divided into subgroups according to the presence of a history of glucocorticoid treatment, disease duration > 15 years and bowel resection. BEV was significantly lower in CD patients with these prevalent risk factors, with no differences in BMD at all sites. Additionally, TBS was reduced in patients with a history of glucocorticoid treatment. Despite a not severely pronounced bone loss in this population, impaired bone quality in CD patients with well-known risk factors for systemic bone loss was assessed by TX-Analyzer™.

## 1. Introduction

Bone loss and increased fracture risk are well-known extraintestinal complications of Crohn’s disease (CD) [1,2]. As has been recently shown by our study group, CD patients have an increased risk for hip fractures and an associated higher mortality risk after fracture compared to the general population [3]. However, overall data on osteoporosis and fracture risk are conflicting in literature and largely depend on the patient population, severity of disease, disease duration and different imaging techniques in assessing bone mineral density (BMD) [4]. Further, patients with CD have many risk factors contributing to bone loss and therefore need special attention to identify patients at risk and prevent fractures. Bone strength is not only determined by BMD, but also by bone microarchitecture. To date, the gold-standard for examination of BMD is dual X-ray absorptiometry (DXA), a two-dimensional imaging method. With respect to the methodology of this imaging technique, no information on bone microstructure is assessed. Furthermore, anterior–posterior DXA scanning may not accurately reflect true changes in BMD, e.g., due to calcification of the aorta or osteophytes within the region of interest (ROI) [5,6,7].

Trabecular bone score (TBS), a grey-level texture parameter, can be applied to lumbar spine DXA scans as an add-on tool and provides information on the trabecular network of vertebral bodies. Additionally, TBS does not seem to be affected by degenerative changes of lumbar spine in contrast to BMD [8].

To date there is only one study addressing TBS assessment in adult CD patients in the literature. Interestingly, no differences in BMD or TBS compared to controls in the total cohort of CD patients were detected, but TBS was decreased in patients with a severe course of disease while BMD showed no difference [9]. Another method for assessing bone microarchitecture in vivo even more precisely is high-resolution peripheral quantitative computed tomography (HR-pQCT) of the distal radius and tibia. Bone microarchitecture assessed by HR-pQCT in inflammatory bowel disease (IBD) patients showed that CD patients have a severe deterioration of cortical and trabecular bone despite a reduced volumetric BMD at the distal radius. In this study, female sex, the diagnosis of CD, lower body mass index (BMI) and a lack of disease remission were identified as independently associated factors with bone loss in IBD [10].

The implementation of novel imaging techniques for the assessment of bone microarchitecture into clinical practice is of major interest. Fractal analysis techniques are based on the fractal model by Mandelbrot [11] and can be used to measure and express complex structures in numeric dimensions and for distinguishing image textures. Extensions of this model led to different techniques examining bone structure from two-dimensional X-ray projections. Fractal analysis of calcaneus radiographs in patients with previous osteoporotic fractures allowed to distinguish patients with fractures from those without fractures, independently and more exactly than BMD [12,13]. In another study, Caligiuri P et al. identified patients with vertebral fractures more accurately using fractal analysis of radiographs of the spine compared to DXA [14].

These results support the hypothesis, that fractal analysis provides complementary information out of conventional radiographs and can improve fracture risk evaluation, independently of BMD and additional radiation exposure.

TX-Analyzer™ is a novel software for fractal-based analysis of radiographs and features three texture algorithms—bone structure value (BSV), bone variance value (BVV) and bone entropy value (BEV). To date, the primary use is for research purposes on different skeletal sites. Until now, only one study on lumbar spine using TX-Analyzer™ was performed. In this retrospective analysis of a large randomized trial in postmenopausal women treated with the monoclonal antibody denosumab over eight years, Dimai HP et al. reported an increase of BMD and BSV of lumbar spine [15].

The aim of the present pilot study was to evaluate bone microstructure assessed by TX-Analyzer™ of the thoracic and lumbar spine in CD patients and for the first time, in the general population to correlate and compare the parameters to standard imaging techniques such as DXA and TBS.

## 2. Patients and Methods

### 2.1. Patients and Study Design

A total number of 40 patients and 40 age and sex-matched controls were analyzed in this case-controlled study. All patients participated in the “Crohn-Bone-Study”, a non-interventional, prospective, cross-sectional, single-center study with the aim to create a database of patients with Crohn’s disease and assess the effects on bone. The patients were recruited in the gastroenterology outpatient clinic of the 1st Medical Department of the Hanusch Hospital of the Austrian Health Insurance in Vienna. Inclusion criteria were a histologically verified diagnosis of CD and patients had to be at least 18 years of age. Out of 51 patients included in the Crohn-Bone-Study, 40 patients underwent DXA scans of the lumbar spine and the hip and radiographs of the thoracic and lumbar spine and were, therefore, selected for this pilot study. Demographic parameters, information on disease duration (DD), treatment including glucocorticoid (GC) therapy, conventional or biological immunosuppressive therapy and history of surgery were assessed. Laboratory results including serum C-reactive protein (CRP), and bone turnover markers including calcium, phosphate, alkaline phosphatase (AP), beta-crosslaps, osteocalcin, parathyroid hormone (PTH) and 25(OH)-vitamin D levels as well as levels of fecal calprotectin were analyzed. Laboratory assessment was performed using immunoturbidimetric assay for CRP, photometric color test for calcium, photometric UV test for phosphate, 2-site immunometric (sandwich) electrochemiluminescence detection assay for beta-crosslaps, chemiluminescence immunoassay for osteocalcin, PTH, 25(OH)-vitamin D and calprotectin. The lower limits of quantification were as follows: 0.2 mg/L for CRP, 0.01 mmol/L for calcium, 0.11 mmol/L for phosphate, 5 U/L for AP, 0.01 ng/mL for beta-crosslaps, 3 ng/mL for osteocalcin, 1 pg/mL for parathyroid hormone, 11 nmol/L for 25(OH)-vitamin D and <5 mg/kg for fecal calprotectin.

Selection of controls was randomly chosen with respect to available conventional radiographs of the thoracic and lumbar spine. In total, 81 subjects who underwent X-ray examinations of the spine at the Radiological Department of the Hanusch Hospital Vienna were screened. Healthy controls were than matched 1:1 to CD, based on age- and sex-distribution of CD. Exclusion criteria for controls were a documented medical history of osteoporosis, low traumatic fractures, diabetes mellitus type 1 or 2, renal insufficiency CKD III-V (chronic kidney disease), hepatic cirrhosis (CHILD B or C), chronic alcohol abuse, rheumatologic disease, malignancy within 5 years or any eating disorder.

Two patients were excluded from the analysis (1 CD and 1 control) due to degenerative changes on the lumbar spine resulting into inaccurate BMD and TX lumbar spine values. In addition, both patients had extremely outranged high BMI (>41 kg/m^2^) and (i) no DXA software for obese patients was available, (ii) TBS cannot be interpreted appropriately in patients with BMI > 40 and (iii) the influence of severe obesity on TX values is unclear.

The final analytical sample therefore comprised 39 CD patients and 39 controls. The study flow-chart is shown in supplementary Figure 1.

The study was approved by the local ethical committee (EK-16-252-1216) and conducted in accordance with the Declaration of Helsinki. All patients agreed to participate to the study and signed an informed consent form.

#### 2.1.1. TX-Analyzer™

The software TX-Analyzer™ (ImageBiopsy Lab., Vienna, Austria) determines structural three-dimensional information of bone architecture non-invasively using a fractal-based analysis out of two-dimensional plain radiographs [16,17]. The software features the three unitless texture algorithms BSV, BVV and BEV. To date, no normative values for these parameters are available.

In detail, BSV reflects the quantification of the fractal dimension of the bone texture via an implementation of a maximum-likelihood estimator of the Hurst coefficient. The Hurst coefficient represents a measure of long-range dependency of data and therefore a good descriptor of wide range of natural phenomena [12,18,19]. This Hurst coefficient was measured in vertical and horizontal directions, for further analysis mean BSV was used. BVV quantifies the Hurst coefficient via the variance of pixel-intensity differences in four directions (0, 45, 90 and 135 degrees) and therefore, the extracted values include the vertical, horizontal and diagonal as well as the mean value, which was further analyzed. BSV and BVV range between 0 and 1; the maximum value of 1 indicates the highest possible homogeneity. The BEV represents the Shannon entropy originating from the field information theory and quantifies the average information content and complexity of the ROI [20].

The analysis of all radiographs was performed by two trained investigators (SH, JES). All X-rays were obtained using Philips DigitalDiagnost.

**Figure 1:**
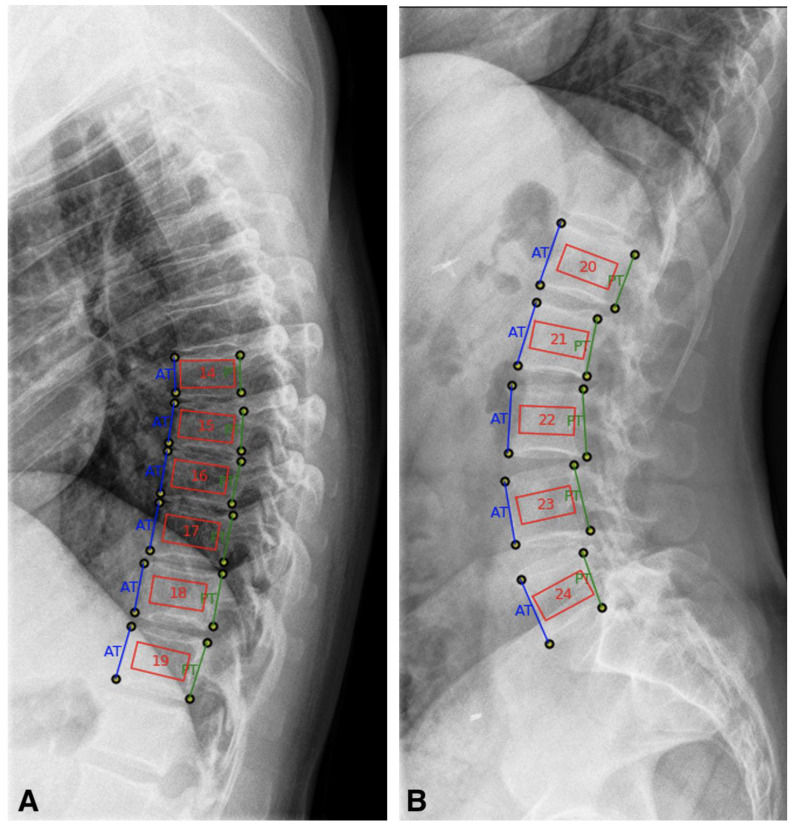
Application of TX-Analyzer™ software on lateral-view radiographs of the thoracic and lumbar spine of patients with Crohn’s disease and controls. Definition of region of interest (ROI) using a preset biopsy mask, after definition of the upper and lower and the anterior and posterior corner of the vertebral bodies the ROI box is placed by the software. (**A)** thoracic spine and (**B)** lumbar spine.

DICOM files of lateral-view radiographs of the thoracic and lumbar spine were analyzed using a semi-automatic software application (IB Lab TX-Analyzer™, IBL, Vienna, Austria). The annotation of the radiographs was carried out and the region of interest was defined. For positioning and analysis of the ROI the preset biopsy mask was used, according to the manufacturers’ recommendation. After marking the front and back corners of each vertebral body, the software places the ROI within the vertebral body. For each subject, all vertebral bodies from the seventh thoracic vertebral body to the fourth lumbar vertebral body were analyzed (Figure 1). Fractured vertebral bodies were excluded from analysis and the mean of the remaining parameters calculated. If only one vertebral body was eligible for analysis, the patient was excluded.

#### 2.1.2. Dual X-ray Absorptiometry (DXA) and Trabecular Bone Score (TBS)

DXA scanning and analysis was performed of the lumbar spine, the total femur and the femoral neck using GE Healthcare Lunar Prodigy (GE©, Boston, MA, USA). BMD and *T*-scores at all three sites were assessed. Analysis of DXA scans was performed according to international guidelines [21] and non-evaluable vertebral bodies excluded, accordingly. If only one vertebral body was evaluable, the measurement was excluded. TBS analysis was performed using TBS iNsight software version 3.

#### 2.1.3. Statistics

Characteristics of CD patients and controls were described using frequencies and percentages for categorical variables and medians and interquartile ranges (IQR) for continuous variables if not stated otherwise. We assessed the distribution of each parameter via normality plots and Kolmogorov–Smirnov test. For group comparisons we used *T*-test or Mann–Whitney *U* test for continuous variables and Chi-square test for categorical variables, as appropriate. *p*-values were two sided, and the statistical significance level was set at 0.05. In case of multiple comparisons, we applied a Bonferroni correction.

For exploration of an association between two continuous variables we calculated either Pearson’s correlation coefficient or Spearman’s rank correlation coefficient, according to the normality of variable distribution. When both variables were normally distributed, we used Pearson’s correlation coefficient, otherwise we used Spearman’s correlation coefficient.

We stratified CD patients by glucocorticoid use (≥5 mg prednisolone-equivalent daily > 3 months users vs. non-users), the disease duration (below or above 15 years), the history of bowel resection (yes vs. no) and compared these groups in demographic, DXA and TX parameters. Due to the study design the selection of healthy controls was solely based on the availability of X-rays, therefore no data of DXA parameters or TBS were available.

In order to test the robustness of our findings, we performed a sensitivity analysis by excluding patients with extreme values for TX parameters and repeated all the analysis. Extreme outliers were defined as values below and above the interquartile range multiplied by 3 (Q1 – 3 × IQR and Q3 + 3 × IQR).

All statistical analyses were conducted in IBM SPSS Statistics for Windows, Version 26 [22].

## 3. Results

### 3.1. Patient Population and Demographic Characteristics

Table 1 summarizes patient characteristics of CD patients and controls. There were no significant differences between CD patients and controls in age, sex or BMI. The disease duration was 8.0 (18) years and the median calprotectin level 88.9 (221.9) mg/kg.

Fifty-nine percent of patients had a history of glucocorticoid treatment > 5 mg daily over more than 3 months. In total, 55.3% of CD patients were treated with conventional disease modifying immunosuppressive drugs (cDMARDs; 6-mercaptopurine, azathioprine, budesonide or mesalazine) and 52.6% with biological DMARDs therapy (bDMARDs; adalimumab, infliximab, ustekinumab, vedolizumab). Laboratory results showed no increase in CRP levels or alterations of bone turnover markers. The median level of 25(OH)-vitamin D in CD patients was 64 (24) nmol/L.

### 3.2. Bone Mineral Density and Bone Microarchitecture

Overall median T-score in lumbar spine of CD patients was −1.4 (2.2) and BMD 1.030 (0.2) g/cm^2^. At the total hip and femoral neck, the BMD was 0.921 (0.13) g/cm^2^ and 0.885 (0.15) g/cm^2^, respectively, with a corresponding T-score of −0.9 (1.2) and −0.9 (1.3). TBS of CD patients was 1.307 (0.2).

At the lumbar spine the TX analysis revealed no differences of BSV, BVV or BEV between CD and controls. BSV and BVV of the thoracic spine were higher in CD patients compared to control patients (*p* = 0.016 and 0.012). All results are summarized in Table 2.

### 3.3. Correlation of Imaging Parameters

All results of correlation analysis are reported in Table 3. BSV, BVV and BEV were correlated with each other within the region of investigation—in lumbar spine BSV and BVV we observed a strong positive correlation (r = 0.800, *R*^2^ = 0.88, *p* < 0.001) and for BSV and BEV a moderate positive correlation (r = 0.660, *p* < 0.001). Comparable significant correlations were found for TX parameters at the thoracic spine. In contrast to the correlations within the two regions, no correlations of TX parameters between the thoracic and the lumbar spine were observed.

BSV, BVV and BEV of the thoracic spine showed no correlation to demographic parameters. At the lumbar spine, for BSV and BVV moderate negative correlations with weight (r = −0.647, *p* < 0.001 and r = −0.605, *p* < 0.001) and BMI (r = −0.568, *p* < 0.001 and r = −0.403, *p* < 0.001) were observed. No significant correlations were found between the TX parameters and TBS.

Further, correlations of TX parameters and BMD at all three measuring sites with laboratory results presented in Table 1 were performed. No correlation was found between TX parameters or BMD with the level of 25(OH)-vitamin D. BSV, BVV and BEV at the lumbar spine were moderately correlated with calprotectin levels (r = 0.438, *p* = 0.009; r = 0.458, *p* = 0.006; and r = 0.518, *p* < 0.001). A negative moderate correlation was observed for BSV and BVV with PTH (r = −0.465, *p* = 0.004 and r = −0.524, *p* = 0.001), but not for BEV. All results are presented in a Supplemental Table S1.

### 3.4. Glucocorticoid Use in Crohn’s Disease Patients

Patients with a GC intake > 3 months had a significantly lower TBS of lumbar spine compared to patients without long-term GC treatment (*p* = 0.014). All further DXA parameters revealed no difference between these two groups.

TX analysis showed a significantly reduced BEV of the thoracic spine in GC treated patients, with no difference in BSV and BVV. At the lumbar spine no difference in BSV, BVV or BEV was found. All parameters are summarized in Table 4.

### 3.5. Disease Duration in Crohn’s Disease Patients

Of the CD patients, 14 patients had a DD of >15 years and 25 patients a DD of ≤15 years. Patients with longer disease duration showed no difference in BMD of the lumbar spine or hip. Further, no difference in TBS was found compared to patients with a shorter disease duration. TX analysis revealed a significantly decreased BEV of the thoracic spine in patients with a DD of >15 years (*p* = 0.001). At the lumbar spine no difference of BEV between the two groups was observed. BSV and BVV showed no differences at the thoracic and the lumbar spine (Table 4).

### 3.6. History of Bowel Resection in Crohn’s Disease Patients

Of the CD patients, 14 patients had a history of surgery, 34.2% of these patients with a resection of the terminal ileum, 10.5% had small bowel resection and 13.2% had a segmental colonic resection—some of the patients had joint procedures therefore the numbers do not sum up. The group of patients with a history of surgery included more male patients, patients were taller (*p* = 0.016) and had a longer disease duration (*p* = 0.013). BMD at the lumbar spine and hip showed no difference between the two groups of CD patients. Additionally, TBS was the same within these two groups. Regarding TX parameters, again BEV was significantly lower in patients with a history of bowel resection (*p* = 0.011), in this group at the lumbar spine. No further differences in TX parameters have been found (Table 4).

### 3.7. Sensitivity Analysis

For sensitivity analyses six patients were excluded with extreme values for TX parameters. A total of 34 CD patients and 38 controls were analyzed. The results with regard to differences between CD and controls in TX parameters remained practically unchanged (Supplemental Table S2). Concerning the analysis of CD patients there were no differences in DXA measures between patients with disease duration below or above 15 years, neither in patients with or without bowel resection. A more pronounced deterioration of bone microarchitecture in GC treated patients represented by lower levels of BSV and BVV at the lumbar spine was observed.

## 4. Discussion

In this pilot study spinal bone microstructure of Crohn’s disease patients and controls of the general population was assessed for the first time using TX-Analyzer™, a novel fractal-based analysis. BSV and BVV of the thoracic spine were higher in CD patients compared to controls, with no difference in BEV. However, a significant impact of risk factors, like disease duration, glucocorticoid treatment and a history of bowel resection, on TX parameters was found in CD patients.

Patients with CD have multiple risk factors for bone loss like malnutrition, inflammatory state, malabsorption associated with vitamin D deficiency and glucocorticoid treatment. In literature, data on osteoporosis and fracture risk in CD patients are incongruent depending on patient population, disease duration and differences in imaging techniques [4]. The BMD measurement of CD patients in the present cohort using DXA technique, the gold standard in clinical practice, revealed overall no severe bone loss. Additional, trabecular bone assessed by TBS showed overall, that only a partially degraded microstructure at the lumbar spine of CD patients. The fact that BSV and BVV showed higher levels of CD patients compared to healthy controls is conflicting, but this should be considered as a result of an overall group, not severely affected bone of the present CD cohort. A recent large retrospective analysis of 393 CD patients revealed that only 19.8% of patients were diagnosed with osteoporosis, whereas the rest had normal BMD (39.9%) or the diagnosis of osteopenia (40.2%). Reduced BMD was associated with risk factors like male sex, low BMI and a history of bowel resection and longitudinal evaluation showed a further reduction of BMD in GC treated patients [23]. These findings indicate that by a modern management of CD using anti-inflammatory medications bone loss overall is not severe, but patients with one or even multiple risk factors and especially those treated with GC treatment should be identified and evaluated.

Further, in the present study, a fractal-based analysis of the spine using TX-Analyzer™ in the general population was performed for the first time. To date no reference values on TX parameters of the spine are available. Due to the study design, no information on BMD or TBS of the control group was available and therefore a deterioration of bone assessed by standardized measurements cannot be ruled out. However, a previous study of Dimai HP et al. focused on a prospective analysis of BSV at the lumbar spine in postmenopausal women on antiresorptive treatment with denosumab and an additional increase of BSV to a gain of BMD was observed [15]. Comparing the presented absolute levels of BSV in this population of women with postmenopausal osteoporosis to those in the present study, levels of BSV and BMD of patients with postmenopausal osteoporosis were much lower compared to CD patients and controls in the present cohort. This supports further the hypothesis of an overall not severely pronounced bone loss in the presented cohort.

It is important to note, that after dividing the population according to three above-mentioned risk factors, significant changes of bone microstructure assessed by TX parameters have been observed. Patients with GC treatment in the medical history, with long standing disease duration and with a history of bowel resection showed a reduction of BEV compared to controls despite no differences in BMD. This highlights that despite an overall not severely pronounced bone loss, deterioration of bone microstructure is assessable at an early time point using BEV.

Additionally, in patients with GC use, this finding is further supported by a reduction of TBS. Interestingly, no differences in BMD were observed. To date, there is only one study assessing TBS in adult CD patients in the literature. Krajcovicova A et al. showed that CD patients with severe disease course showed a reduction of TBS while BMD remained the same as controls [9]. These findings are in accordance with the present findings, since patients treated repeatedly with GC over time can be assumed to have a more severe course of disease.

However, long-term GC treatment itself is a well-known risk factor for bone loss due to inhibition of calcium absorption and promotion of renal calcium loss [24,25] and a promotion of osteoblast apoptosis and decreasing levels of osteoprotegerin [26,27]. Further, significant alterations of bone microarchitecture in patients treated with GC have been previously described in literature [28,29]. In a study of Leib ES et al. TBS was significantly lower in patients treated with GC due to different diseases compared to patients without GC treatment, while areal BMD at the lumbar spine showed no difference. Therefore, the authors stated, that GC use is associated with an impairment of microarchitectural texture of the central skeleton measured by TBS, preceding changes of BMD [30].

Deteriorations of bone microstructure in CD patients have been described previously in the literature. HR-pQCT scans of CD patients in a tertiary care center showed a severe deterioration of cortical and trabecular bone despite a reduced volumetric BMD of the ultradistal radius [10]. Nevertheless, those patients overall had a more severe course of disease compared to the present cohort and for the clinical setting this method has a limitation due to its rare availability. The most accurate information on bone microstructure is gained by performing a transiliac bone biopsy with histomorphometry. In a study by Oostlander AE et al. transiliac bone biopsies of 23 CD patients in remission were analyzed and histomorphometric analysis showed a reduction in bone mass characterized by trabecular thinning, caused by reduced bone formation [31]. All these studies provide important information on the risk of trabecular bone loss in patients with CD. Due to the limitations of availability and invasiveness, new imaging tools such as TX-Analyzer™ are of major interest for the clinical setting since pre-existing radiographs can be analyzed without additional radiation exposure.

TBS is the only available texture parameter in clinical practice to gain additional information on bone microstructure out of DXA scans. Spinal osteoarthrosis has no significant effect on TBS, while BMD increases in contrast [8]. Therefore, imaging techniques assessing bone microstructure within the vertebral body are of major interest. Since lower TBS as well as BEV values in the patient population with prior GC treatment despite normal BMD levels were found, one can suggest, that a fractal-based analysis using TX-Analyzer™ may as well elucidate early degradation of bone microstructure, prior to a loss of BMD and irrespectively of degenerative spinal changes. The acquisition of conventional radiographs due to different reasons is much more widespread compared to the performance of DXA scans, especially in younger patients. Therefore, TX-Analyzer™ may identify patients at risk at an early time point and before fractures even occur.

This pilot study is not without limitations. The main limitation is, that the size of the cohort was overall relatively small and therefore not suitable for building a regression model to address the question if two or further risk factors contribute to trabecular bone loss. Further studies with bigger study populations are required to confirm our preliminary results.

Additional, correlation analysis showed a positive correlation of lumbar and thoracic TX parameters, but no correlation between the different areas. Further, since there is a negative correlation observed of BSV, BVV and BEV of the lumbar spine with demographic parameters like height, weight and BMI, while there were no correlations with thoracic spine parameters, an influence of visceral fat on results of the lumbar spine may be a potential explanation. Furthermore, these results have to be further investigated and validated in a bigger cohort, since the present patient population and controls was overall well balanced and did not meet the recommendations of the World Health Organization for nutrition state with a median BMI of 27 kg/m^2^. Nevertheless, taking this limitation into account, BEV reduction at the thoracic spine was present in two subgroups, despite an overall not reduced BMD. Further, validated imaging methods like TBS were lower in GC treated patients in addition to low BEV at the thoracic spine, supporting the findings. To date only limited information and experience with the TX-Analyzer™ at the spine is available and no reference values of the general population have been assessed previously. Therefore, we were able to demonstrate data on TX parameters of the thoracic and lumbar spine in healthy subjects for the first time.

## 5. Conclusions

In conclusion, the software TX-Analyzer™ is a non-invasive tool for indirect assessment of bone microstructure based on existing conventional radiographs. In the present study, CD patients were not severely affected by systemic bone loss and therefore, no typical microstructure pattern by this method was assessable compared to controls. However, well-known risk factors for systemic bone loss like glucocorticoid treatment, disease duration and history of bowel resection resulted in impaired bone quality assessed by TX-Analyzer™. Especially patients with GC treatment showed pronounced changes in BEV in accordance with a reduction of TBS despite normal BMD assessed by DXA.

## Figures and Tables

**Table 1:** Demographics and disease specific characteristics of patients with Crohn‘s disease (CD) and controls.

	Crohn’s Disease *n* = 39	Controls *n* = 39	*p*
Demographics and Disease Duration			
Sex [male/female]	13/26	14/25	0.812
Age [years]	53.9 (40.4–60.7)	44.8 (41.3–55.9)	0.439
Height [m]	1.68 (1.6–1.74)	1.67 (1.62–1.72)	0.859
Weight [kg]	75 (59–94)	78 (64.8–85.3)	0.895
BMI [kg/m^2^]	27.4 (22.1–32.3)	27.1 (23.4–30.2)	0.924
Laboratory Results			
CRP [mg/L, 0.0–4.9]	2.80 (1.40–7.30)	-	-
Ca [mmol/L, 2.20–2.65]	2.37 (2.31–2.53)	-	-
Ph [mmol/L, 0.81–1.45]	1.14 (1.00–1.27)	-	-
AP [U/L, 30–120]	78 (65–95)	-	-
Beta-crosslaps [ng/mL, 0.03–0.37]	0.390 (0.28–0.58)	-	-
PTH [pg/mL, 12–88]	45.5 (31–73)	-	-
Osteocalcin [ng/mL, 4.6–65.4]	16.5 (14.1–21.9)	-	-
25(OH)-vitamin D [nmol/L, 75–250]	64 (52–76)	-	-
Calprotectin [mg/kg, 0.0–50.0]	88.9 (26.1–248)	-	-
Disease Characteristics			
Disease duration [years]	8 (2–20)	-	-
cDMARDs, *n* (%)	21 (55.3)	-	-
bDMARDs, *n* (%)	20 (52.6)	-	-
GC-Treatment > 3 mo, *n* (%)	23 (59)	-	-
History of bowel resection due to CD, *n* (%) *	14 (35.9) *		
Ileocoecal Resection, *n* (%)	13 (34.2)	-	-
Small bowel resection, *n* (%)	4 (10.5)	-	-
Colonic resection, *n* (%)	5 (13.2)	-	-

Notes: BMI, body mass index; laboratory results [unit, reference range]; CRP, C-reactive protein; Ca, calcium; Ph, phosphate; AP, alkaline phosphatase; PTH, parathyroid hormone; cDMARDs, conventional disease modifying immunosuppressive drugs (including 6-mercaptopurine, azathioprine, budesonide or mesalazine); bDMARDs, biologic disease modifying immunosuppressive drugs (including adalimumab, infliximab, ustekinumab, vedolizumab); GC-treatment > 3 mo, glucocorticoid treatment over 3 months ≥ 5 mg; * some patients had joint procedures therefore the numbers do not sum up; and all parameters are reported as median (25th–75th percentile) or number (%), respectively, level of significance *p* < 0.05.

**Table 2:** Results of TX analysis, dual X-ray absorptiometry (DXA) and trabecular bone score (TBS) of patients with Crohn‘s disease and controls.

	Crohn’s Disease *n* = 39	Controls *n* = 39	*p*
*DXA and TBS*			
Lumbar Spine (L1–L4)			
BMD [g/cm^2^]	1.030 (0.955–1.183)	-	-
T-Score [SD]	−1.4 (−2.1–0.1)	-	-
TBS [units]	1.307 (1.218–1.402)	-	-
Total Hip			
BMD [g/cm^2^]	0.921 (0.847–0.978)		-
T-Score [SD]	−0.9 (−1.4, −0.2)		-
Femoral Neck			
BMD [g/cm^2^]	0.885 (0.787–0.938)	-	-
T-Score [SD]	−0.9 (−1.8, −0.5)	-	-
*TX Analysis*			
Thoracic Spine (T7–T12)			
BSV	0.293 (0.268–0.309)	0.270 (0.225–0.302)	0.016
BVV	0.282 (0.260–0.302)	0.251 (0.214–0.288)	0.012
BEV	12.2 (12.1–12.3)	12.2 (12.1–12.3)	0.919
Lumbar Spine (L1–L4)			
BSV	0.109 (0.093–0.128)	0.110 (0.104–0.125)	0.310
BVV	0.100 (0.092–0.118)	0.103 (0.010–0.119)	0.301
BEV	11.5 (11.4–11.6)	11.5 (11.4–11.6)	0.154

Notes: BMD, bone mineral density; SD, standard deviation; BSV, bone structure value; BVV, bone variance value; BEV, bone entropy value; BSV, BVV and BEV are unitless; and all parameters are reported as median (25th–75th percentile), level of significance *p* < 0.05.

**Table 3:** Correlations of demographics and imaging parameters assessed by TX analysis, bone mineral density and trabecular bone score.

	Age	Weight	Height	BMI	BSV T7–T12	BSV L1–L4	BVV T7–T12	BVV L1–L4	BEV T7–T12	BEV L1–L4	BMD L1–L4	BMD Fem.Neck	BMD Total Fem
Weight	0.220												
Height	0.151	0.498 **											
BMI	0.159	0.876 **	0.025										
BSV T7–T12	0.029	−0.046	0.032	−0.078									
BSV L1–L4	−0.200	−0.647 *	−0.359 **	−0.568 **	0.109								
BVV T7–T12	0.054	−0.093	0.070	−0.151	0.966 **	0.134							
BVV L1–L4	−0.304 **	−0.605 **	−0.250 *	−0.594 **	0.152	0.800* *	0.196						
BEV T7–T12	−0.086	−0.082	−0.142	−0.019	0.476 **	0.041	0.349 **	−0.003					
BEV L1–L4	−0.154	−0.476 **	−0.259 *	−0.403 **	0.060	0.666 **	0.047	0.452 **	0.151				
BMD L1–L4	−0.095	0.407 *	0.204	0.360 *	0.014	−0.250	−0.121	−0.134	0.417 **	0.115			
BMD fem.neck	−0.273	0.454 **	0.337 *	0.353 *	0.010	−0.314	−0.097	−0.122	0.349 *	−0.029	0.785 **		
BMD total fem	−0.120	0.545 **	0.256	0.507 **	−0.064	−0.472 **	−0.177	−0.264	0.358 *	−0.238	0.771 **	0.881 **	
TBS	−0.525 **	0.154	−0.037	0.191	−0.102	−0.067	−0.165	0.067	0.217	0.116	0.417 *	0.466 **	0.451 **

Note: fem, femoral; and * level of significance *p* < 0.05; ** level of significance *p* < 0.001, (with adjustment for a Bonferroni correction). Values in bold are considered as statistically significant also after Bonferroni correction.

**Table 4:** Analysis of TX parameters, DXA and TBS in patients distributed according to disease duration, glucocorticoid (GC) use and history of bowel resection.

	History of GC Treatment		*p*	Disease Duration		*p*	History for Surgery for CD		*p*
	<3 months *N* = 16	>3 months *N* = 23		≤15 years *N* = 25	> 15 years *N* = 14		No *N* = 25	Yes *N* = 14	
*Demographics and Disease Duration*									
Sex [male/female]	5/11	8/15	0.818	6/19	7/7	0.098	4/21	9/5	0.002
Age [years]	44.4 (35.9–56.4)	54.8 (41.8–61.4)	0.228	54.3 (36.0–62.6)	52.7 (42.1–56.9)	0.883	53.9 (38.8–58.6)	50.8 (39.9–62.6)	0.972
Height [m]	1.65 (1.56–1.7)	1.69 (1.63–1.74)	0.579	1.66 (1.59–1.7)	1.70 (1.62–1.78)	0.268	1.67 (1.59–1.7)	1.74 (1.65–1.79)	0.016
Weight [kg]	70 (55-94.8)	80 (61–90)	0.346	75 (60.5–94.5)	73 (49.8–89.3)	0.519	73 (59–85)	88.5 (57.8–105.5)	0.111
BMI [kg/m^2^]	26.3 (21.7–32.9)	28.3 (22.7–31.6)	0.908	28.3 (23.1–32.6)	26.7 (18.2–30.3)	0.170	26.6 (22.3–32.0)	29.2 (21.5–32.4)	0.613
Dis. duration [years]	6.5 (2–13)	12 (5–25)	0.053	5 (1.5–8)	22 (17.8–30.5)	< 0.001	5 (1.5–14)	17 (8–22.8)	0.013
*DXA and TBS*									
Lumbar Spine (L1–L4)									
BMD [g/cm^2^]	1.062 (0.994–1.282)	0.976 (0.912-1.183)	0.145	1.086 (0.972–1.223)	0.963 (0.874–1.047)	0.337	1.036 (0.952–1.223)	1.029 (0.919–1.076)	0.567
*T*-Score [SD]	−1.2 (−1.6–0.8)	−1.8 (−2.2–0.1)	0.146	−1.1 (−1.8–0.3)	−1.9 (−2.6–1.4)	0.412	−1.2 (−2.0–0.3)	−1.5 (−2.3–1.2)	0.357
TBS [units]	1.397 (1.299–1.459)	1.257 (1.186–1.356)	0.014	1.336 (1.222–1.403)	1.274 (1.210–1.390)	0.361	1.302 (1.202–1.399)	1.323 (1.240–1.407)	0.527
Total Hip									
BMD [g/cm^2^]	0.921 (0.895–1.040)	0.922 (0.814–0.978)	0.251	0.943 (0.886–1.024)	0.855 (0.770–0.931)	0.598	0.921 (0.849–0.990)	0.926 (0.838–1.003)	0.942
*T*-Score [SD]	−0.8 (−1.5–0.3)	−1.1 (−1.7–0.2)	0.159	−0.6 (−1.1–0.2)	−1.4 (−2.3–0.6)	0.425	−0.8 (−1.3–0.1)	−1.2 (−1.6–0.3)	0.443
Femoral Neck									
BMD [g/cm^2^]	0.900 (0.813–1.015)	0.857 (0.766–0.938)	0.224	0.900 (0.829–0.943)	0.813 (0.757–0.947)	0.724	0.885 (0.786–0.936)	0.901 (0.781–1.015)	0.536
*T*-Score [SD]	−0.8 (−1.5–0.3)	−1.0 (−1.8–0.6)	0.215	-0.8 (−1.3–0.5)	-1.4(−2.3–0.6)	0.373	−0.8 (−1.7–0.5)	−1.1 (−2.1–0.3)	0.844
*TX Analysis*									
Thoracic Spine (T7–T12)									
BSV	0.295 (0.270–0.305)	0.290 (0.259–0.315)	0.932	0.298 (0.269–0.308)	0.284 (0.266–0.313)	0.761	0.293 (0.269–0.308)	0.284 (0.266–0.314)	0.919
BVV	0.275 (0.260–0.294)	0.286 (0.243–0.310)	0.475	0.282 (0.251–0.303)	0.278 (0.255–0.309)	0.806	0.276 (0.251–0.303)	0.286 (0.255–0.304)	0.828
BEV	12.3 (12.2–12.4)	12.2 (12.1–12.3)	0.015	12.3 (12.2–12.4)	12.1 (12.0–12.2)	0.001	12.2 (12.1–12.3)	12.2 (12.1–12.4)	0.784
Lumbar Spine (L1–L4)									
BSV	0.115 (0.093–0.131)	0.103 (0.096–0.126)	0.797	0.109 (0.093–0.126)	0.106 (0.093–0.176)	0.478	0.115 (0.096–0.132)	0.100 (0.090–0.117)	0.228
BVV	0.107 (0.092–0.118)	0.099 (0.086–0.122)	0.549	0.099 (0.091–0.118)	0.105 (0.091–0.146)	0.515	0.099 (0.092–0.124)	0.101 (0.086–0.115)	0.675
BEV	11.6 (11.4–11.6)	11.5 (11.3–11.6)	0.138	11.5 (11.4–11.6)	11.5 (11.3–11.6)	0.303	11.6 (11.5–11.6)	11.4 (11.3–11.5)	0.011

Note: Dis.duration, disease duration; BSV, BVV and BEV are unitless; and all parameters are reported as median (25th–75th percentile), level of significance *p* < 0.05.

## Supplementary Materials

The following are available online at https://www.mdpi.com/2077-0383/9/12/4116/s1, Figure S1: Study flow-chart, Table S1: Correlations of imaging parameters assessed by TX analysis, Bone Mineral Density and Trabecular Bone Score and laboratory results, Table S2: Analysis of TX parameters, DXA and TBS in patients distributed according to disease duration, GC use and history of bowel resection after exclusion of extreme outliers.

## References

[1] Targownik L.E., Bernstein C.N., Nugent Z., Johansson H., Oden A., McCloskey E., Kanis J.A., Leslie W.D. (2013). Inflammatory bowel disease and the risk of fracture after controlling for FRAX. J. Bone Miner. Res..

[2] van Staa T.P., Cooper C., Brusse L.S., Leufkens H., Javaid M.K., Arden N.K. (2003). Inflammatory bowel disease and the risk of fracture. Gastroenterology.

[3] Bartko J., Reichardt B., Kocijan R., Klaushofer K., Zwerina J., Behanova M. (2020). Inflammatory Bowel Disease: A Nationwide Study of Hip Fracture and Mortality Risk After Hip Fracture. J. Crohn’s Colitis.

[4] Targownik L.E., Bernstein C.N., Nugent Z., Leslie W.D. (2013). Inflammatory bowel disease has a small effect on bone mineral density and risk for osteoporosis. Clin. Gastroenterol. Hepatol..

[5] Drinka P.J., DeSmet A.A., Bauwens S.F., Rogot A. (1992). The effect of overlying calcification on lumbar bone densitometry. Calcif. Tissue Int..

[6] Orwoll E.S., Oviatt S.K., Mann T. (1990). The Impact of Osteophytic and Vascular Calcifications on Vertebral Mineral Density Measurements in Men*. J. Clin. Endocrinol. Metab..

[7] Greenspan S.L., Maitland-Ramsey L., Myers E. (1996). Classification of osteoporosis in the elderly is dependent on site-specific analysis. Calcif. Tissue Int..

[8] Dufour R., Winzenrieth R., Heraud A., Hans D., Mehsen N. (2013). Generation and validation of a normative, age-specific reference curve for lumbar spine trabecular bone score (TBS) in French women. Osteoporos. Int. J. Establ. Result Coop. Eur. Found. Osteoporos. Natl. Osteoporos. Found. USA.

[9] Krajcovicova A., Kuzma M., Hlavaty T., Hans D., Koller T., Jackuliak P., Leskova Z., Sturdik I., Killinger Z., Payer J. (2018). Decrease of trabecular bone score reflects severity of Crohn’s disease: Results of a case-control study. Eur. J. Gastroenterol. Hepatol..

[10] Haschka J., Hirschmann S., Kleyer A., Englbrecht M., Faustini F., Simon D., Figueiredo C.P., Schuster L., Muschitz C., Kocijan R. (2016). High-resolution Quantitative Computed Tomography Demonstrates Structural Defects in Cortical and Trabecular Bone in IBD Patients. J. Crohn’s Colitis.

[11] Mandelbrot B.B., Freeman W.H. (1983). The Fractal Geometry of Nature.

[12] Benhamou C.L., Poupon S., Lespessailles E., Loiseau S., Jennane R., Siroux V., Ohley W., Pothuaud L. (2001). Fractal analysis of radiographic trabecular bone texture and bone mineral density: Two complementary parameters related to osteoporotic fractures. J. Bone Miner. Res..

[13] Pothuaud L., Lespessailles E., Harba R., Jennane R., Royant V., Eynard E., Benhamou C.L. (1998). Fractal analysis of trabecular bone texture on radiographs: Discriminant value in postmenopausal osteoporosis. Osteoporos. Int..

[14] Caligiuri P., Giger M.L., Favus M. (1994). Multifractal radiographic analysis of osteoporosis. Med. Phys..

[15] Dimai H.P., Ljuhar R., Ljuhar D., Norman B., Nehrer S., Kurth A., Fahrleitner-Pammer A. (2019). Assessing the effects of long-term osteoporosis treatment by using conventional spine radiographs: Results from a pilot study in a sub-cohort of a large randomized controlled trial. Skeletal. Radiol..

[16] Pentland A.P. (1984). Fractal-based description of natural scenes. IEEE Trans. Pattern Anal. Mach. Intell..

[17] Nehrer S., Ljuhar R., Steindl P., Simon R., Maurer D., Ljuhar D., Bertalan Z., Dimai H.P., Goetz C., Paixao T. (2019). Automated Knee Osteoarthritis Assessment Increases Physicians’ Agreement Rate and Accuracy: Data from the Osteoarthritis Initiative. Cartilage.

[18] Prouteau S., Ducher G., Nanyan P., Lemineur G., Benhamou L., Courteix D. (2004). Fractal analysis of bone texture: A screening tool for stress fracture risk?. Eur. J. Clin. Investig..

[19] Lundahl T., Ohley W.J., Kay S.M., Siffert R. (1986). Fractional brownian motion: A maximum likelihood estimator and its application to image texture. IEEE Trans. Med. Imaging.

[20] Shannon C.E. (1997). The mathematical theory of communication. 1963. MD Comput..

[21] 2019 ISCD Official Positions—Adult. https://www.iscd.org/official-positions/2019-iscd-official-positions-adult/.

[22] IBM Corp. (2019). Released 2019. IBM SPSS Statistics for Windows, Version 26.0.

[23] Hoffmann P., Krisam J., Kasperk C., Gauss A. (2019). Prevalence, Risk Factors and Course of Osteoporosis in Patients with Crohn’s Disease at a Tertiary Referral Center. J. Clin. Med..

[24] Suzuki Y., Ichikawa Y., Saito E., Homma M. (1983). Importance of increased urinary calcium excretion in the development of secondary hyperparathyroidism of patients under glucocorticoid therapy. Metab. Clin. Exp..

[25] Hahn T.J., Halstead L.R., Baran D.T. (1981). Effects off short term glucocorticoid administration on intestinal calcium absorption and circulating vitamin D metabolite concentrations in man. J. Clin. Endocrinol. Metab..

[26] Kondo T., Kitazawa R., Yamaguchi A., Kitazawa S. (2008). Dexamethasone promotes osteoclastogenesis by inhibiting osteoprotegerin through multiple levels. J. Cell. Biochem..

[27] Adami G., Saag K.G. (2019). Glucocorticoid-induced osteoporosis: 2019 concise clinical review. Osteoporos. Int..

[28] Dalle Carbonare L., Arlot M.E., Chavassieux P.M., Roux J.P., Portero N.R., Meunier P.J. (2001). Comparison of trabecular bone microarchitecture and remodeling in glucocorticoid-induced and postmenopausal osteoporosis. J. Bone Miner. Res. Off. J. Am. Soc. Bone Miner. Res..

[29] Dempster D.W. (1989). Bone histomorphometry in glucocorticoid-induced osteoporosis. J. Bone Miner. Res. Off. J. Am. Soc. Bone Miner. Res..

[30] Leib E.S., Winzenrieth R. (2016). Bone status in glucocorticoid-treated men and women. Osteoporos. Int. J. Establ. Result Coop. Eur. Found. Osteoporos. Natl. Osteoporos. Found. USA.

[31] Oostlander A.E., Bravenboer N., Sohl E., Holzmann P.J., van der Woude C.J., Dijkstra G., Stokkers P.C., Oldenburg B., Netelenbos J.C., Hommes D.W. (2011). Histomorphometric analysis reveals reduced bone mass and bone formation in patients with quiescent Crohn’s disease. Gastroenterology.

