# Supplementary material for: Fractal-Based Analysis of Bone Microstructure in Crohn’s Disease: A Pilot Study
Source: J Clin Med. 2020 Dec 20;9(12):4116. doi: 10.3390/jcm9124116 (PMC7766043; doi:10.3390/jcm9124116)
Supplement: Supplementary file 1 [file jcm-09-04116-s001.pdf]

Supplementary Materials: Figure S1: Results imaging parameters according to disease duration, GC use and history of bowel resection after exclusion of extreme outliers

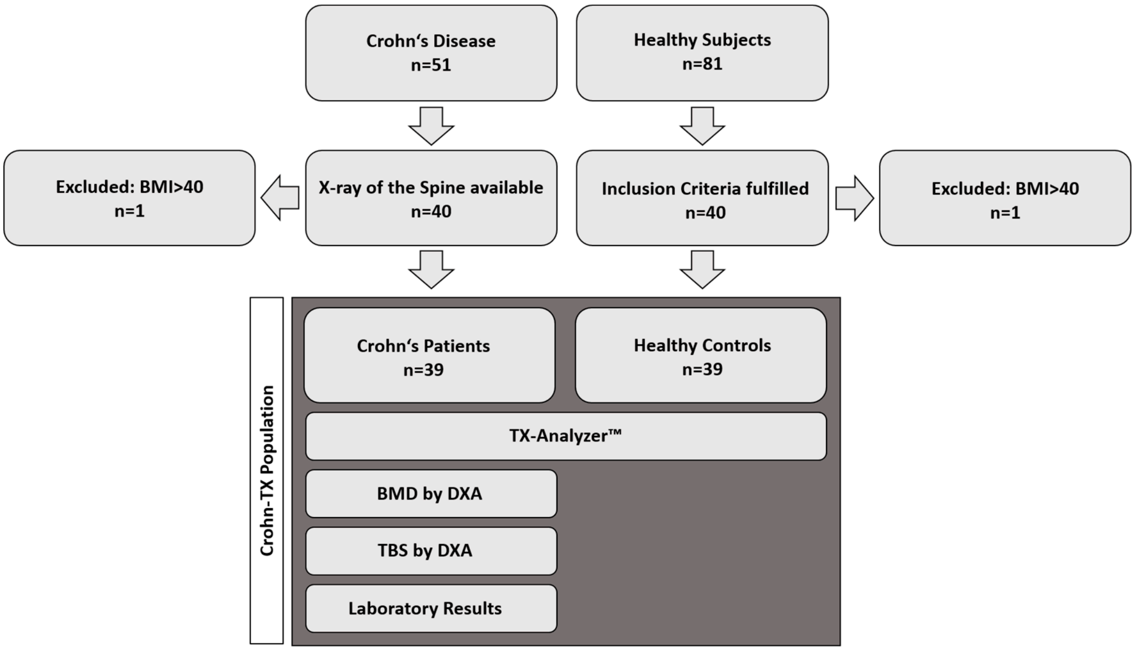

Figure S1. Study flow-chart. Healthy controls were matched 1:1 to Crohn's Disease (CD) patients, based on age- and sex-distribution. Two subjects (1 CD, 1 CTRL) were excluded because of their inaccurate BMD, TX and BMI, since (i) no DXA software for obese patients was available, (ii) TBS cannot be interpreted appropriately in patients with BMI>40 and (iii) the influence of severe obesity on TX-values is unclear. For TX-analysis 39 CDs and 39 controls were included. Bone mineral density (BMD) and trabecular bone score (TBS) by Dual Energy X-ray Absorptiometry (DXA) as well as laboratory results were available in CD only.

Table S1. Correlations of imaging parameters assessed by TX analysis, Bone Mineral Density and Trabecular Bone Score and laboratory results.

	**CRP**	**Ca**	**Ph**	**AP**	**PTH**	**Osteocalcin**	**Beta-Crosslaps**	**25(OH)Vitamin D**	**Calprotectin**
	**[mg/l]**	**[mmol/l]**	**[mmol/l]**	**[U/l]**	**[pg/ml]**	**[ng/ml]**	**[ng/ml]**	**[nmol/l]**	**[mg/kg]**
**BSV T7-T12**	-0.080	-0.112	0.001	0.019	-0.233	-0.358*	-0.266	-0.044	-0.001
**BSV L1-L4**	-0.042	0.062	0.236	-0.236	**-0.465****	-0.111	-0.141	-0.037	**0.438****
**BVV T7-T12**	-0.086	-0.048	-0.001	0.134	-0.194	-0.234	-0.181	-0.013	0.026
**BVV L1-L4**	-0.013	0.069	0.088	0.011	**-0.524****	-0.149	-0.190	0.092	**0.458****
**BEV T7-T12**	0.086	-0.257	-0.016	-0.032	-0.097	-0.352*	-0.267	-0.093	0.182

**BEV L1-L4**	0.136	0.098	0.243	-0.210	-0.298	-0.092	0.067	-0.125	**0.518****
**BMD L1-L4 [g/cm²]**	0.224	-0.114	0.106	0.165	0.076	-0.071	-0.161	-0.150	0.134
**BMD fem.neck [g/cm²]**	0.194	-0.018	0.136	0.218	0.028	-0.065	-0.027	-0.212	0.149
**BMD Total fem [g/cm²]**	0.233	-0.091	-0.001	0.246	0.160	0.081	-0.019	-0.186	0.026
**TBS [units]**	0.265	-0.176	-0.125	0.024	0.147	-0.070	-0.067	-0.032	0.142

CRP, C-reactive protein; Ca, Calcium; Ph, Phosphate; AP, Alkaline Phosphatase; PTH, parathyroid hormone; BSV, Bone Structure Value; BVV, Bone Variance Value; BEV, Bone Entropy Value; BSV, BVV and BEV are unitless; BMD, Bone Mineral Density; TBS, Trabecular Bone Score; *level of significance $p < 0.05$; ** level of significance $p < 0.001$.

Table S2. Analysis of TX parameters, DXA and TBS in patients distributed according to disease duration, GC use and history of bowel resection after exclusion of extreme outliers.

	**History of GC Treatment**		***p***	**Disease Duration**		***p***	**History for surgery for CD**		***p***
	<3 months *n*=15	>3 months *n*=19		<15 years *n*=24	>15 years *n*=10		No *n*=22	Yes *n*=12	
Demographics and Disease Duration									
Sex [male/female]	4/11	7/12	0.818	5/19	4/6	**0.026**	3/19	8/4	**0.002**
Age [years]	42.3 (20.4)	55.5 (19.6)	0.175	54.3 (23.7)	46.9 (16.3)	0.838	54.1 (21.9)	44.9 (21.1)	0.765
Height [m]	1.64 (0.15)	1.69 (0.11)	0.060	1.66 (0.11)	1.71 (0.10)	0.081	1.67 (0.1)	1.74 (0.1)	**0.005**
Weight [kg]	70 (40)	84 (24)	0.153	74.0 (32.8)	80.5 (32.0)	0.756	73.0 (24.3)	88.5 (44.8)	0.195
BMI [kg/m²]	26.2 (10.9)	28.4 (6)	0.409	27.9 (9.6)	28 (10)	0.591	27.0 (9.2)	29.2 (11.4)	0.898
Disease duration	6 (11)	9 (16)	0.159	4.5 (7)	21 (9)	**<0.001**	4.5 (9)	17 (14)	**0.004**
DXA and TBS									
<u>Lumbar Spine (L1 – L4)</u>									
BMD [g/cm²]	1.036 (0.333)	1.046 (0.258)	0.352	1.074 (0.267)	1.018 (0.242)	0.323	1.090 (0.284)	1.029 (0.103)	0.471
T-Score [SD]	-1.2 (2.8)	-1.3 (2.3)	0.319	-1.0 (2.1)	-1.6 (2.2)	0.208	-0.8 (2.3)	-1.5 (0.9)	0.283
TBS [units]	1.392 (0.169)	1.257 (0.166)	**0.035**	1.335 (0.182)	1.289 (0.189)	0.847	1.335 (0.194)	1.289 (0.186)	0.928
<u>Total Hip</u>									
BMD [g/cm²]	0.921 (0.174)	0.937 (0.161)	0.428	0.944 (0.152)	0.907 (0.173)	0.222	0.930 (0.144)	0.944 (0.205)	0.828
T-Score [SD]	-0.8 (1.7)	-0.6 (1.2)	0.345	-0.6 (1.4)	-1.2 (1.5)	0.073	-0.7 (1.3)	-1.0 (1.4)	0.265

				<u>Femoral Neck</u>					
BMD [g/cm²]	0.914 (0.179)	0.900 (0.151)	0.369	0.905 (0.090)	0.882 (0.241)	0.724	0.893 (0.106)	0.920 (0.281)	0.471
T-Score [SD]	-0.8 (1.0)	-0.9 (1.4)	0.304	-0.8 (0.6)	-1.2 (2.1)	0.373	-0.8 (0.8)	-0.9 (2.1)	0.922
				TX Analysis					
				<u>Thoracic Spine (T7 – T12)</u>					
BSV	0.293 (0.04)	0.290 (0.06)	0.953	0.297 (0.04)	0.283 (0.04)	0.537	0.293 (0.04)	0.283 (0.06)	0.769
BVV	0.274 (0.03)	0.274 (0.06)	0.878	0.279 (0.06)	0.269 (0.05)	0.647	0.274 (0.05)	0.275 (0.06)	0.651
BEV	12.3 (0.2)	12.2 (0.1)	0.127	12.3 (0.2)	12.1 (0.2)	**0.033**	12.3 (0.2)	12.2 (0.2)	0.286
				<u>Lumbar Spine (L1 – L4)</u>					
BSV	0.115 (0.04)	0.101 (0.03)	**0.048**	0.112 (0.03)	0.101 (0.08)	0.198	0.110 (0.03)	0.100 (0.02)	0.310
BVV	0.109 (0.03)	0.094 (0.02)	**0.042**	0.099 (0.03)	0.096 (0.02)	0.284	0.097 (0.02)	0.101 (0.03)	0.929
BEV	11.6 (0.2)	11.5 (0.2)	0.052	11.5 (0.2)	11.5 (0.2)	0.190	11.5 (0.1)	11.4 (0.2)	**0.032**

	**History of GC Treatment**		***p***	**Disease Duration**		***p***	**History for surgery for CD**		***p***
	< 3 months *n*=15	> 3 months *n*=19		< 15 years *n*=24	> 15 years *n*=10		No *n*=22	Yes *n*=12	
				Demographics and Disease Duration					
Sex [male/female]	4/11	7/12	0.818	5/19	4/6	**0.026**	3/19	8/4	**0.002**
Age [years]	42.3 (20.4)	55.5 (19.6)	0.175	54.3 (23.7)	46.9 (16.3)	0.838	54.1 (21.9)	44.9 (21.1)	0.765
Height [m]	1.64 (0.15)	1.69 (0.11)	0.060	1.66 (0.11)	1.71 (0.10)	0.081	1.67 (0.1)	1.74 (0.1)	**0.005**
Weight [kg]	70 (40)	84 (24)	0.153	74.0 (32.8)	80.5 (32.0)	0.756	73.0 (24.3)	88.5 (44.8)	0.195
BMI [kg/m²]	26.2 (10.9)	28.4 (6)	0.409	27.9 (9.6)	28 (10)	0.591	27.0 (9.2)	29.2 (11.4)	0.898
Disease duration	6 (11)	9 (16)	0.159	4.5 (7)	21 (9)	**<0.001**	4.5 (9)	17 (14)	**0.004**
				DXA and TBS					
				<u>Lumbar Spine (L1 – L4)</u>					
BMD [g/cm²]	1.036 (0.333)	1.046 (0.258)	0.352	1.074 (0.267)	1.018 (0.242)	0.323	1.090 (0.284)	1.029 (0.103)	0.471
T-Score [SD]	-1.2 (2.8)	-1.3 (2.3)	0.319	-1.0 (2.1)	-1.6 (2.2)	0.208	-0.8 (2.3)	-1.5 (0.9)	0.283
TBS [units]	1.392 (0.169)	1.257 (0.166)	**0.035**	1.335 (0.182)	1.289 (0.189)	0.847	1.335 (0.194)	1.289 (0.186)	0.928
				<u>Total Hip</u>					
BMD [g/cm²]	0.921 (0.174)	0.937 (0.161)	0.428	0.944 (0.152)	0.907 (0.173)	0.222	0.930 (0.144)	0.944 (0.205)	0.828
T-Score [SD]	-0.8 (1.7)	-0.6 (1.2)	0.345	-0.6 (1.4)	-1.2 (1.5)	0.073	-0.7 (1.3)	-1.0 (1.4)	0.265
				<u>Femoral Neck</u>					
BMD [g/cm²]	0.914 (0.179)	0.900 (0.151)	0.369	0.905 (0.090)	0.882 (0.241)	0.724	0.893 (0.106)	0.920 (0.281)	0.471

T-Score [SD]	-0.8 (1.0)	-0.9 (1.4)	0.304	-0.8 (0.6)	-1.2 (2.1)	0.373	-0.8 (0.8)	-0.9 (2.1)	0.922
TX Analysis									
Thoracic Spine (T7 – T12)									
BSV	0.293 (0.04)	0.290 (0.06)	0.953	0.297 (0.04)	0.283 (0.04)	0.537	0.293 (0.04)	0.283 (0.06)	0.769
BVV	0.274 (0.03)	0.274 (0.06)	0.878	0.279 (0.06)	0.269 (0.05)	0.647	0.274 (0.05)	0.275 (0.06)	0.651
BEV	12.3 (0.2)	12.2 (0.1)	0.127	12.3 (0.2)	12.1 (0.2)	**0.033**	12.3 (0.2)	12.2 (0.2)	0.286
Lumbar Spine (L1 – L4)									
BSV	0.115 (0.04)	0.101 (0.03)	**0.048**	0.112 (0.03)	0.101 (0.08)	0.198	0.110 (0.03)	0.100 (0.02)	0.310
BVV	0.109 (0.03)	0.094 (0.02)	**0.042**	0.099 (0.03)	0.096 (0.02)	0.284	0.097 (0.02)	0.101 (0.03)	0.929
BEV	11.6 (0.2)	11.5 (0.2)	0.052	11.5 (0.2)	11.5 (0.2)	0.190	11.5 (0.1)	11.4 (0.2)	**0.032**

GC treatment, glucocorticoid treatment; CD, Crohn's Disease; DXA, Dual X-ray absorptiometry; TBS, Trabecular Bone Score; BMD, Bone Mineral Density; SD, Standard Deviation; BSV, Bone Structure Value; BVV, Bone Variance Value; BEV, Bone Entropy Value; BSV, BVV and BEV are unitless; all parameters are reported as median (IQR), level of significance $p < 0.05$.
